# Effect of Flavonoid Supplementation on Alveolar Bone Healing—A Randomized Pilot Trial

**DOI:** 10.3390/dj8030086

**Published:** 2020-08-04

**Authors:** Jose Moises Souza, Stephen A. Tuin, Adam G. Robinson, Joao Gustavo Oliveira de Souza, Marco Aurelio Bianchini, Patricia A. Miguez

**Affiliations:** 1 Centro de Ciências da Saúde, Departamento de Odontologia, Campus Reitor David Ferreira Lima, Universidade Federal de Santa Catarina, Bairro Trindade, Florianópolis 88040-970, Brazil; zeh_3112@hotmail.com (J.M.S.J.); joaogustavo_s@hotmail.com (J.G.O.d.S.); bian07@yahoo.com.br (M.A.B.); 2 Oral and Craniofacial Health Sciences, Adams School of Dentistry, Koury Oral Health Sciences Building, Rm 4608, CB# 7455, University of North Carolina at Chapel Hill, 385 South Columbia Street, Chapel Hill, NC 27599-7455, USA; satuin@gmail.com (S.A.T.); agrobins@live.unc.edu (A.G.R.); 3 Division of Comprehensive Oral Health, Adams School of Dentistry, Koury Oral Health Sciences Building, Rm 4610, CB# 7455, University of North Carolina at Chapel Hill, Chapel Hill, NC 77599-7455, USA

**Keywords:** tooth socket, phytochemicals, collagen, osteoclast, dietary supplements, flavonoid

## Abstract

We investigated the effects of two common dietary supplements on bone healing in dental extraction sockets in humans. In this randomized pilot trial, male subjects took Grape Seed Extract [GSE] or Grapefruit Extract [GFE] starting two weeks prior to dental extraction and maintained this regimen for sixty days after surgery. Extraction sockets were filled with a collagen plug. After 24 h, a socket sample was collected and processed for quantitative real-time reverse transcription polymerase chain reaction (qRT-PCR) and an 84-gene wound healing assay. Sixty days after tooth extraction, a core of newly formed bone was obtained prior to dental implant placement and processed for histology. qRT-PCR revealed that GFE led to a significant decrease in platelet-derived growth factor and interleukin (IL)1-β compared to GSE, and a significant decrease in IL-6 and CXCL2 compared to control. GSE led to a significant increase in coagulation factor Von Willebrand and inflammatory marker IL1-β compared to GFE. WISP1 and CXCL5 were upregulated in both groups. Overall, GFE showed a downregulation of inflammation and GSE led to a decrease in collagen density and increased osteoclasts. This pilot trial highlights the need for further investigation on the mechanism of action of such supplements on bone healing and oral health.

## 1. Introduction

Alveolar healing after dental extraction is of importance in dentistry due to the changes that occur in the alveolar process that may prevent or make it difficult to install a dental implant and/or a prosthesis [1,2]. In recent years, there has been increasing interest in the use of naturally available botanicals as preventive or therapeutic agents for some diseases. Regular intake of such compounds may affect bone homeostasis and bone healing. One popular over-the-counter option for health management is grape seed extract. Proanthocyanidins (PAs) (a class of polyphenols), are the most abundant phenolic compound in grape seeds [3]. PAs have several reported biological functions, including antibacterial, antiviral, anti-inflammatory, anti-oxidant, and vasodilation [4,5,6]. Little is known about the effects of PAs on bone health in general and more specifically on the alveolar process. One study showed that the systemic use of PA increased the expression of mRNA and bone morphogenetic protein (BMP)-7 in diabetic rats [7]. PA has been used for the treatment of rheumatoid arthritis, and led to a decrease in interleukin (IL)-17 expression in rodents [5]. Other studies with PA-treated animals have demonstrated decreased expression of IL-4, IL-5, IL-13, vascular endothelial growth factor A (VEGFA), and transforming growth factor (TGF)-β1 in acute and chronic asthma models [8,9].

Another popular oral supplement is grapefruit extract, which contains mostly naringenin/naringin (NA) and hesperidin (HE), both of which are flavonoid compounds found abundantly in citrus fruits [10,11]. Studies have reported that these compounds elicit anti-tumor, anti-oxidant, anti-inflammatory, and anti-diabetic effects [12,13,14,15,16]. HE derivatives were evaluated for anti-inflammatory activity in a macrophage cell line and acute liver injury model and showed reduction in levels of IL-6 and (tumor necrosis factor)TNF-α in vitro and in vivo [17]. Other studies have demonstrated potential benefits of NA administration in rodent models, including lower levels of IL-4 and IL-13 in an asthma animal model [18], decreased TGF-β1 in a renal injury model [16,19,20], and decreased inflammation of rat skin induced by ultra-violet radiation, which led to the reduction of several interleukins, TNF-α, and TGF-β1 [21,22]. These studies demonstrate that there are several pro-inflammatory, anti-inflammatory, and growth factors that may be modulated by HE and NA. However, the majority of literature to date is focused on the anti-inflammatory properties of flavonoids, including grapefruit extract [23]. Finally, narirutin, another flavanone found in grapefruit extract, has also been investigated in the context of reducing inflammation, although with limited evidence [24].

PAs have also been studied in terms of bone homeostasis. In one study, the results indicated that PA consumption led to an increase in both bone mass and bone strength in the mandibular condyle of rats [25,26]. A study on the effects of PA on tibial diaphysis in rats with low calcium content suggested that PA included in a calcium diet had a beneficial effect on bone formation and bone strength [27]. PA was used in the treatment of osteoarthritis in rat knee joints and protected against further damage by reducing inflammation [28]. PA has also been investigated as a treatment for rheumatoid arthritis in rats. Administration of PA led to improved outcomes for arthritis by simultaneously suppressing osteoclast differentiation and promoting the differentiation of osteoblasts [29]. In vivo, the effect of HE and NA on the regulation of bone metabolism in rats has demonstrated protection against bone loss, acting primarily through inhibition of bone resorption, as well as promotion of osteogenesis [30,31,32].

Self-administration of natural compounds by the general public is on the rise. An emerging strategy for enhancing overall health has been to increase intake of dietary supplements. Data from the 2012 National Health Interview Survey found that non-vitamin and non-mineral natural products are the most popular complementary health methods [33]. Thus, it is important to understand the systemic effects of such compounds in oral health and healing. A better understanding of the effects of such natural compounds, including those derived from fruit extracts, is needed to understand their potential effects on bone physiology and oral health in the context of tissue repair. The aim of this study was to evaluate if commercially available dietary supplements rich in PA, NA, and HE would influence bone healing when taken during healing of dental extraction sockets. We hypothesize that natural products such as grape seed and grapefruit extract will lead to a favorable effect on bone healing due to a positive modulation of inflammation, growth factor levels and collagen matrix deposition. The hypothesis was tested by evaluating if commercially available dietary supplements rich in PA, NA and HE (such as grape seed and grapefruit extract) when taken during dental extraction socket healing would influence bone parameters as measured by gene expression and histomorphometric analyses.

## 2. Materials and Methods

### 2.1. Subject Recruitment, Randomization and Inclusion/Exclusion Criteria

The study was a double-blind, prospective randomized pilot trial to evaluate the effect of two dietary supplements on the clinical healing of bone in dental extraction sockets at 24 h and sixty days post-extraction. The study was approved by the National Commission for Research Ethics (CONEP 740.532) in Brazil on 8 August 2014 for adult male patients who required the extraction of non-restorable single-rooted teeth and installation of oral dental implants for prosthetic rehabilitation. The trial was registered at the Brazilian National Registry for Clinical Trials (Registro Brasileiro de Ensaios Clinicos, ReBec, Rio de Janeiro, Brazil): RBR-56q7h9. Exclusion criteria included history of chemotherapy and radiotherapy of the head and neck, previous periodontal disease, alcohol abuse, bisphosphonate therapy, non-controlled diabetes, smokers, and/or individuals who presented with any contraindication to oral surgery. Female patients were not included to minimize variations in healing due to gender related hormone differences. Patients from 21 to 70 years of age (mean 48.1 y.o.) were screened at the dental clinic of the Center for Dental Implant Studies (CEPID) of the Department of Dentistry of the Federal University of Santa Catarina. All patients signed a Free and Informed Consent Term authorizing their participation and collection of clinical and histological specimens. Participants were randomly assigned to three groups as follows:

- GROUP 1 (GSE): Grape Seed Extract (rich in Proanthocyanidins)
- GROUP 2 (GFE): Grapefruit Extract (rich in Naringenin/Hesperidin)
- GROUP 3 (CON): Control (No phytochemical)

**Figure 1:**
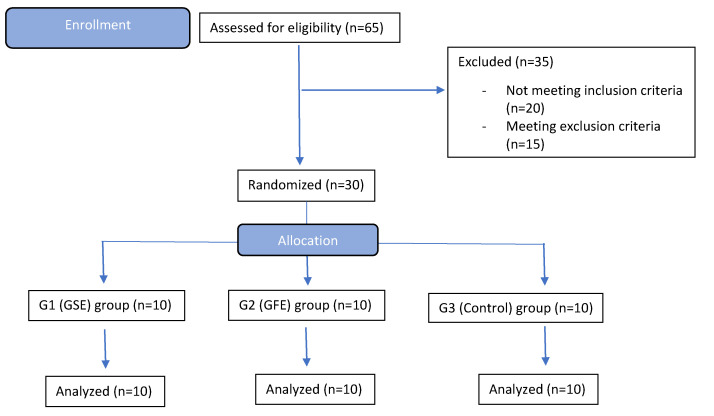
CONSORT flow diagram of enrollment, randomization, and treatment. None of the allocated patients withdrew from the study.

A sample size calculation was performed based on the protocol described in sealedenvelope.com (Sealed Envelope Ltd., London, UK) using the trial equivalent with alpha = 5%, power 95%. Each treatment modality included 10 patients per group among 30 patients that qualified for the study (out of 65 patients screened). Patients were randomly assigned to one of the three treatment groups by a study coordinator of CEPID according to a random allocation protocol. The primary health care provider at CEPID had no access to the allocation sequence. Outcomes were assessed by a second health care provider and researchers at the University of North Carolina with no access to any patient identification records (stored at CEPID, Federal University of Santa Catarina). All patients successfully completed the study as depicted in the CONSORT flow diagram (Figure 1). Patients in the GSE group took 300 mg/day (Grape Seed Extract, GNC, Pittsburg, PA, USA) and patients in the GFE group 250 mg t.i.d. (Grapefruit Extract, Nature’s Way, Springville, UT, USA) as recommended by the manufacturers. Patients in the control group received no phytochemicals prior to and after surgical interventions. Patients started taking the supplements with a generalized label (not identifying the compound) fifteen days prior to dental extraction surgery and maintained this regimen for another sixty days after surgery, totaling seventy-five days of use (10 weeks). Continuous use of supplements was confirmed through interviews before the surgical procedure and post-operative consultations as well as review of a take-home diary which included confirmation of intake of supplements, and any changes in diet or consumption of more than one serving of items rich in phytochemicals such as chocolate, wine, tea and coffee. The blinded clinical center health care provider was instructed to collect information on all adverse events defined as “Any unfavorable and unintended sign, symptom, or disease temporally associated with the use of a medicinal product, whether or not considered related to the medicinal product” (International Conference on Harmonisation of Technical Requirements for Registration of Pharmaceuticals for Human Use, Guideline for Industry: Clinical Safety Data Management: Definitions and Standards for Expedited Reporting, March 1995). None of the patients withdrew from the study or reported any adverse event. The surgeon was blind to the treatment assignment, as was the examiner of the samples for gene and histological analyses. The CONSORT checklist and trial protocol are available as Supplementary Materials (S1 CONSORT checklist, S1 Protocol). The initial protocol included another treatment group with a phytochemical extract from gardenia. However, since only the powder form was available for purchase over the counter, this supplement was not included in the study to avoid variability in dosing. Further, the final number of subjects enrolled was 30, due to difficulty in identifying eligible patients.

### 2.2. Surgical Procedures

#### 2.2.1. Dental Extraction and Sample Collection from the Alveolus

**Figure 2:**
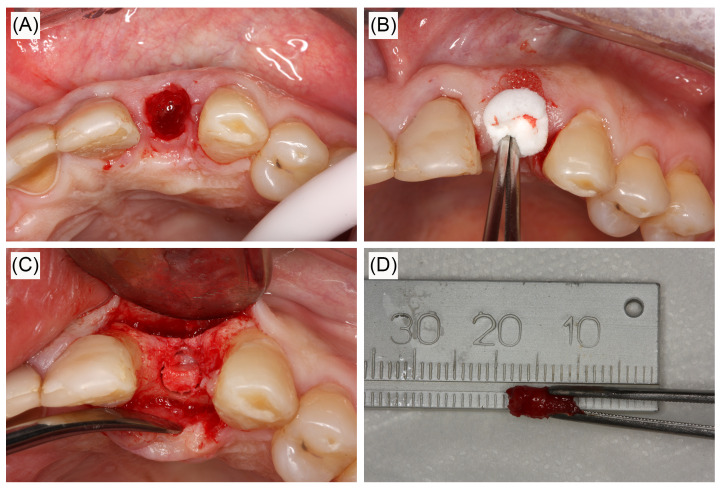
Clinical figures representative of alveolar sockets and sample collection in the study. (**A**) Extraction socket. (**B**) Collagen sponge in socket. Sponges were collected after 24 h for RNA extraction and quantitative real-time reverse transcription polymerase chain reaction (qRT-PCR) analysis. (**C**,**D**) Central core removed via trephine bur 60 days post-extraction for histological analyses.

Tooth extractions were carried out in atraumatic manner. The extraction site was filled with a collagen sponge (Collagen Plug, Zimmer, Carlsbad, CA, USA) and sutured with braided silk 4-0 (Silk 4-0 Ethicon, Johnson & Johnson, São Paulo, SP, Brazil) (Figure 2A,B). Participants were instructed in post-operative care, including pain management with only acetaminophen 750 mg t.i.d. for five days. Twenty-four hours after the extraction of the tooth, all patients returned for collection of biological material for gene analysis (removal of the collagen plug with blood clot formed within the alveolus). This time point for collection was chosen based on evidence that inflammatory cells and clot stability would be present at 24 h [2]. The biopsy specimen was immediately placed in an RNA stabilization solution (RNAlater, Ambion, Austin, TX, USA) and frozen. All patients had a second post-surgery follow up within seven days of the first procedure to ensure healing was within normal limits.

#### 2.2.2. Bone Biopsy

Sixty days after dental extraction, a full thickness flap was lifted and a cylindrical bone specimen of 3 × 6 mm was obtained via a trephine drill prior to site preparation before implant placement. Between 6–8 weeks post-extraction, the newly formed bone is deposited and starts to go through remodeling which would allow full characterization of effect of dietary supplements on initial bone healing [2,34]. The bone samples were collected and fixed in paraformaldehyde for 3 days at 4 °C and then transferred to saline solution and stored at 4 °C until use (Figure 2C,D).

### 2.3. Tissue Sample Analyses

#### 2.3.1. Quantitative Real Time Reverse Transcription Polymerase Chain Reaction (qRT-PCR) of Individual Genes and Wound Healing Array

Isolated RNA was purified using a TRIzol Plus RNA Purification kit (ThermoFisher Scientific, Waltham, MA, USA) according to the manufacturer’s instructions. One µL of purified RNA was analyzed with a NanoDrop One^C^ (Thermo Scientific) to determine RNA yield and cDNA libraries were generated using an Omniscript Reverse Transcriptase Kit (Qiagen, Valencia, CA, USA) and Oligo(dT)_12-18_ primers (ThermoFisher) according to the manufacturer’s instructions. qRT-PCR was performed in triplicate using one µL of cDNA and TaqMan Gene Expression Master Mix with uracil-N-glycosylase (UNG) (Thermo Scientific). TaqMan primers (Thermo Scientific) for the following individual genes were analyzed: TGF-β1 (*TGFB1*), *VEGFA*, PDGF (*PDGFA*), IL-1β (*IL1B*), IL-6 (*IL6*), chemokine (C-X-C motif) ligand 2 (*CXCL2*), von Willebrand factor (*VWF*), and endogenous housekeeping control glyceraldehyde 3-phosphate dehydrogenase (*GAPDH*). These genes were chosen based on studies that evaluated biological molecules of importance on healing of extraction sockets over time [34,35,36]. qRT-PCR reactions were performed on an ABI Prism 7000 sequence detection system (Applied Biosystems, Foster City, CA, USA). Fold change gene expression was calculated using the comparative threshold (C_T_) method [37].

Samples from each group were subjected to RT^2^ Profiler PCR Array analysis, probing for 86 gene targets related to wound healing (Qiagen). Arrays were loaded with samples according to the manufacturer’s instructions and analyzed on an ABI StepOnePlus detection system (Applied Biosystems). Gene expression analysis and statistics were computed using the manufacturer’s RT^2^ Profiler PCR Array Data Analysis Webportal Software (Qiagen).

#### 2.3.2. Histological Staining

The bone cores harvested at 60 days post-extraction were demineralized in 0.5 M EDTA, pH 7.4, for nine weeks at 4 °C. Samples were paraffin embedded and six μm thick serial sections were stained for hematoxylin and eosin (H&E) for gross anatomy, tartrate-resistant acid phosphatase (TRAP) for osteoclast activity, and picrosirius red (PSR) for collagen organization [38,39].

#### 2.3.3. Histological Analyses

H&E samples were imaged at 40× magnification and a composite stitched image of the entire sample was created using Photoshop^®^ software (Adobe^®^ Systems, San Jose, CA, USA). Bone area was computed in ImageJ (NIH, Bethesda, MD, USA).

PSR stitched images were analyzed using a custom generated algorithm in Matlab^®^ R2016a (Mathworks, Natick, MA, USA). The percent area of red, yellow, and green signal was normalized to total color signal for each sample as previously described [40].

Osteoclasts were counted as positive TRAP stained cells that also contained at least three nuclei. The number of bone lining cells was determined to estimate the osteoblast activity of each sample by counting nuclei without TRAP staining residing on bone surfaces [41].

### 2.4. Statistical Analyses

Statistical analyses for bone area, PSR color distribution, cell count, and qRT-PCR of individual genes was performed using a one-way ANOVA and Bonferonni correction post hoc analysis with *p* < 0.05 considered significant with JMP Pro 11 software (SAS, Cary, NC, USA). Wound healing array statistics were computed using the manufacturer’s RT^2^ Profiler PCR Array Data Analysis Webportal Software (Qiagen). An alpha value of <0.05 was used for all tests and Tukey post hoc analysis used to confirm statistical differences.

## 3. Results

### 3.1. qRT-PCR and Wound Healing Array

**Figure 3:**
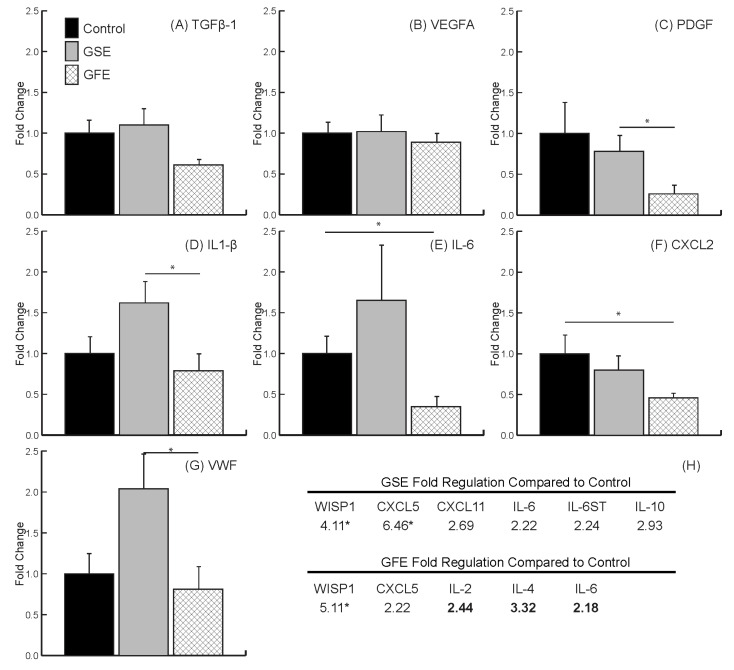
qRT-PCR results. No differences found for transforming growth factor TGF-β1 (**A**) and vascular endothelial growth factor (VEGFA) (**B**). Grapefruit extract (GFE) led to a significant decrease in the growth factor PDGF (**C**), and inflammatory marker IL1-β (**D**) compared to grape seed extract (GSE), and a significant decrease in inflammatory markers IL-6 (**E**) and CXCL2 (**F**) compared to control. GSE led to a significant increase in the coagulation von Willebrand factor (VWF) (**G**), and inflammatory marker IL1-β (**D**) compared to GFE. (**H**) Wound healing PCR array highlighted results. Statistically significant genes indicated by * (*p* < 0.05). Standard text indicates upregulated and bold text indicates downregulated. Bars are SDs.

The individual gene targets analyzed (*TGFB1*, *VEGFA*, *PDGFA*, *IL1B*, *IL6*, *CXCL2* and *VWF*) were chosen due to their importance in early bone healing [36]. *TGFB1* was not statistically downregulated in the GFE group (0.61 ± 0.07) or the GSE group (1.1 ± 0.22) compared to control (Figure 3A) (control values normalized to 1). There was also no significant differences in the expression of *VEGFA* between treatments (1.02 ± 0.18 and 0.89 ± 0.11, GSE and GFE, respectively) compared to control (Figure 3B). Lastly, the expression of *PDGFA* was significantly downregulated in the GFE group compared to the GSE group (0.26 ± 0.07 and 0.78 ± 0.17, GFE and GSE, respectively). However, there was no significant difference in either group compared to control (Figure 3C).

Analysis of inflammation marker genes indicated that treatment with GFE led to a significant downregulation in the expression of *IL1B* (Figure 3D) in the GFE group (0.79 ± 0.17) compared to GSE (1.62 ± 0.27) as well as of *IL6* (Figure 3E) and *CXCL2* (Figure 3F) (0.35 ± 0.1 for *IL6 and* 0.46 ± 0.07 for *CXCL2*) compared to control. Lastly, treatment with GSE led to a significant increase in the expression of coagulation factor *VWF* compared to GFE (2.04 ± 0.44 and 0.81 ± 0.26 for GSE and GFE, respectively) and *IL1B* (1.62 ± 0.27 and 0.79 ± 0.17 for GSE and GFE, respectively) (Figure 3D,G).

Results from the wound healing PCR array indicated that treatment with GSE led to an increase in genes considered as inflammatory markers *CXCL5*, *CXCL11*, *IL6*, *IL6ST*, and *IL10*. In contrast, treatment with GFE only led to an increase in *CXCL5*, with downregulation of *IL2*, *IL4*, and *IL6*. Interestingly, both treatments led to a significant upregulation of the osteogenic marker WNT1-inducible-signaling pathway protein 1 (*WISP1*) (Figure 3H).

### 3.2. Histomorphometric Analysis

**Figure 4:**
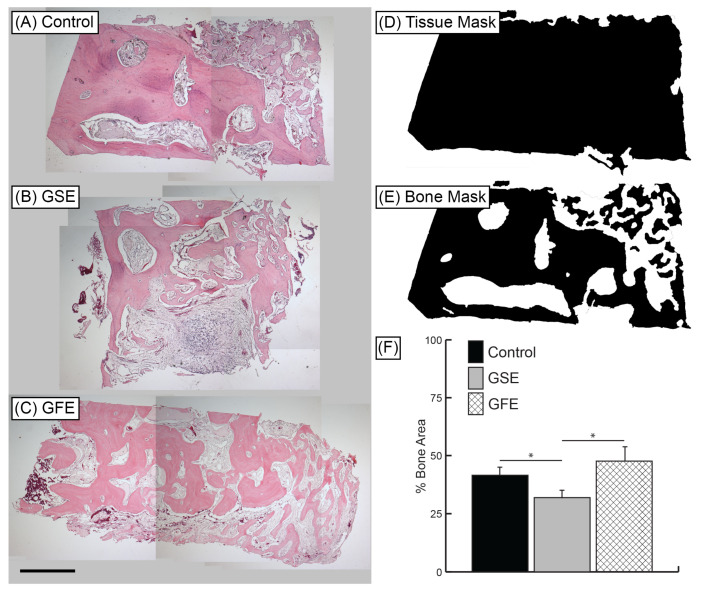
Representative H&E samples at 40× magnification. (**A**) Control group, (**B**) GSE treatment, and (**C**) GFE treatment. Scale bar indicates 1 mm. Histomorphometric analysis: Whole tissue masks (**D**) and bone area masks (**E**) were generated using Adobe^®^ Photoshop^®^ software. Bone area and tissue area were calculated using ImageJ software. (**F**) Average percent bone area. GSE treatment led to significantly reduced bone area compared to control and GFE groups. Statistically significant results indicated by * (*p* < 0.05). Bars are SDs.

H&E histological sections (Figure 4A–C) were quantified using tissue mask technique (Figure 4D,E) and revealed treatment with GSE led to a significant decrease in bone area compared to controls (41.5% ± 4.1% and 31.9% ± 3.0% for control and GSE groups, respectively) (Figure 4F). There was no significant difference in new bone area for the GFE group (47.6% ± 6.3%) compared to control; however, there was a significant increase in bone formation compared to the GSE group (Figure 4F). Qualitative examination of GSE samples also revealed increased amounts of platelets and loose connective tissue compared to control and GFE treated groups (Figure 4A–C).

### 3.3. PSR Quantification

**Figure 5:**
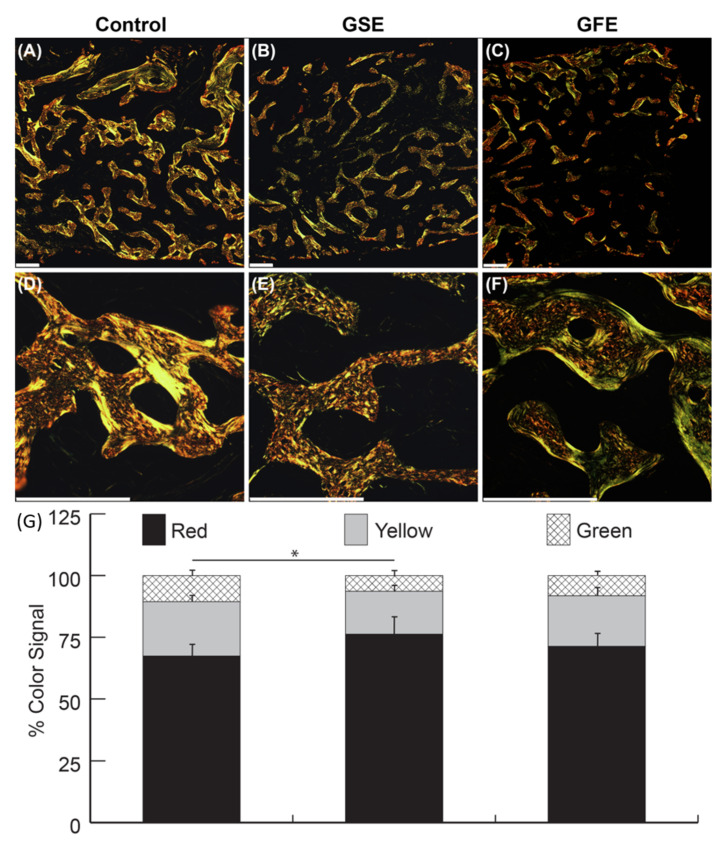
Representative picrosirius red (PSR) samples at 40× magnification (top row), and 200× magnification (middle row). Scale bars indicate 300 μm. Qualitative analysis revealed control (**A**,**D**) had less evidence of new woven bone than GSE samples (**B**,**E**). GFE samples (**C**,**F**) also exhibited a clear pattern of woven bone compared to control group, but not to the extent of GSE treated samples. Control and GFE samples appeared to have more mature lamellar bone in both loose and densely packed structures. Quantitatively, GSE treatment (middle column) led to significantly decreased yellow and green signals, and significantly increased red signal compared to the control group (first column) (**G**). Statistically significant results indicated by * (*p* < 0.05). Bars are SDs.

Qualitative analysis of samples under polarized light revealed control group (Figure 5A,D) showed a different pattern of bone formation compared to treatment groups. There was a greater amount of new woven bone in GSE samples (Figure 5B,E) compared to control (Figure 5A,D). GFE samples (Figure 5C,F) also exhibited greater amounts of woven bone compared to control groups (Figure 5A,D), but not to the extent of GSE treated samples (Figure 5B,E). Control and GFE samples appeared to have more mature lamellar bone in both loose and densely packed structures. Quantitative analysis of color signal distribution of PSR stained samples viewed under polarized light revealed no significant differences with respect to red, yellow, and green signals between the control and GFE treated groups (68.8% ± 5.6% and 72.2% ± 5.6% red, 22.4% ± 3.1% and 20.8% ± 3.4% yellow, 10.8% ± 3.1% and 8.3% ± 2.5% green, for control and GFE, respectively). However, there was a significant difference in color signal distribution in the GSE treated group with increased red signal (74.9% ± 7.4% red, 17.1% ± 3.8% yellow, and 6.3% ± 2.3% green) compared to control (Figure 5G). PSR analyses was calculated in Matlab as previously described [40]. 

### 3.4. Osteoclast Quantification

**Figure 6:**
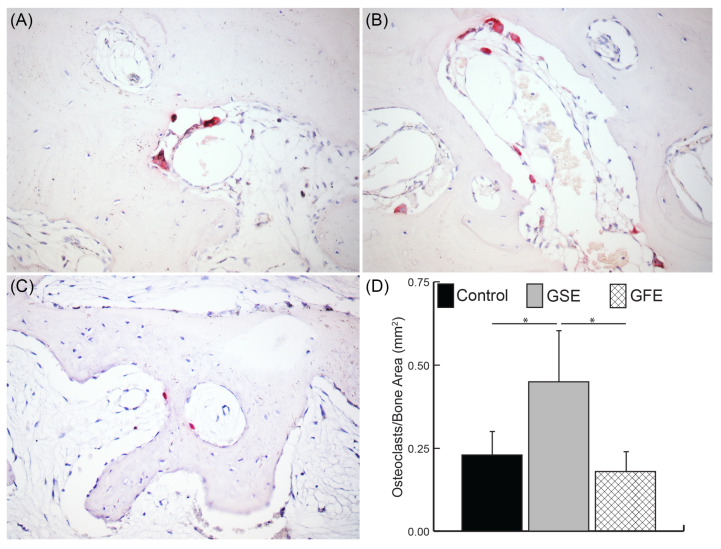
Osteoclast quantification. Sample images of control (**A**), GSE (**B**), and GFE (**C**) shown at 200× magnification. Positive tartrate-resistant acid phosphatase (TRAP) stained cells (red) with at least three nuclei were counted as an osteoclast for the entire sample and normalized to total bone area (**D**). GSE treatment led to a significant increase in osteoclast density compared to control and GFE groups (**D**) Statistically significant results indicated by * (*p* < 0.05). Bars are SDs.

Osteoclasts were counted as multinucleated (more than three nuclei) TRAP+ cells (Figure 6A–C). GSE treatment led to a significant increase in the osteoclast density compared to the control group and the GFE treated group (0.23 ± 0.08, 0.45 ± 0.17, and 0.18 ± 0.05 osteoclasts/mm^2^, for control, GSE, and GFE groups, respectively) (Figure 6D). The GFE treated group exhibited similar osteoclast density to controls. Similarly, while not significant, a greater number of osteoblasts were observed in the GSE group compared to controls (207.6 ± 97.0 and 302.3 ± 157.9 osteoblasts/mm^2^ for control and GSE, respectively), while a decrease in osteoblasts was observed for the GFE group (109.4 ± 30.3 osteoblasts/mm^2^). The ratio of osteoblasts to osteoclasts was calculated for each treatment group as an indicator of extent of remodeling versus resorption. Results indicated that control and GSE groups had similar osteoblast/osteoclast ratios (43.7 ± 16.9 and 50.3 ± 25.1 for control and GSE, respectively), while GFE treatment led to a tendency towards reduction in the osteoblast/osteoclast ratio (22.7 ± 10.7), although these results did not reach significance.

## 4. Discussion

To the authors’ knowledge, this is the first study to analyze the effects of dietary supplements on bone healing in humans. One study investigated not homeostasis but healing of tibia in rats and found that local delivery of HE in a gelatin scaffold in conjunction with mesenchymal cells improved bone regeneration [42].

In the present study, dietary supplementation with GFE, which contains NA and HE, demonstrated a tendency to decrease gene expression of growth factor TGF-β1 in relation to the control group, and statistical decrease in growth factor PDGF in relation to the GSE group. A decrease was also found for the inflammatory marker IL-1β, in relation to the GSE group, while CXCL2 and IL-6 both had a decrease in relation to the control group. The decrease in the level of TGF-β1 has been reported in rat studies for treatment of renal toxicity, pancreatic, and breast cancer [16,19,20,43,44]. A decrease in PDGF has also been reported in rats treated for hepatic fibrosis [15]. The results of inflammatory marker expression corroborate with other studies in rodents [22,45]. These results are in agreement with the literature on the use of grapefruit extract and support an anti-inflammatory effect and a proliferative/formation of connective tissue effect, since these interleukins are critical in the inflammatory process. TGF-β1 and PDGF are of vital importance in bone formation [36,46] but it is difficult to predict the appropriate levels at specific time points since studies vary in their methodologies and have not investigated minimal thresholds needed for healing over time. 

The individuals treated with GSE, which contains proanthocyanins, had an increase in the expression of the coagulation factor VWF in relation to the GFE group. Another in vitro study evaluated the effects of proanthocyanin on the selective inhibition of cell adhesion molecule expression demonstrating a decrease in VWF [47]. However, this study was conducted in vitro and studied the effect on cells under diabetic conditions expressing large amounts of inflammation. An in vivo study observed the effect of GSE in a murine model for vascular thrombosis. It was concluded that the extract reduces thrombosis by decreasing cell adhesion molecules and VWF factor [48]. The increase of VWF factor in the present study may suggest an attempt to achieve better blood coagulation, owing to a greater degree of inflammatory infiltrate and platelets in the socket area for the group treated with GSE. 

The increase in inflammatory factors with grape seed extract does not agree with induced inflammation studies in animals [28,49]. However, these studies evaluated a different healing condition with a larger dose of extract per body mass per day, which likely influenced the cellular response. The large doses used in animals are not currently recommended for human consumption. The doses utilized in this study were as recommended by manufacturers. GSE and GFE are Generally Recognized As Safe (GRAS) by the US Food and Drug Administration (FDA) and tolerated in moderate doses without side effects (Natural Standard database and http://nccam.nih.gov/health/grapeseed/ataglance). In the present study, none of the enrolled participants reported adverse effects.

Histomorphometric analysis showed a statistically significant reduction of bone/total tissue in the GSE group compared to the control group. Other studies in rats that have evaluated the effect of proanthocyanin supplementation in bone homeostasis, showed increased bone mass, resistance, and bone formation [25,26,27,50,51]. However, direct comparisons are difficult as all of these in vivo studies examined different conditions using animal models, and differing dose and disease models.

A qualitative change in collagen deposition pattern was observed in the GSE group compared to the GFE and control groups, as evidenced by less compacted fibers viewed histologically. Previous studies have reported that PSR color correlates with collagen fiber thickness, with thicker, densely packed collagen appearing red, intermediate thicknesses appearing yellow, and thin loosely packed collagen appearing green [40]. A color shift from green to red is typically observed over time in soft tissue that could be attributed to maturity [52]. However, in the present study, native mature bone appears green, with newly formed loosely packed woven bone appearing red. In the present study, the thickest and most packed collagen in fully mineralized areas was green, possibly indicative of collagen content typically found in fully mineralized tissues [53]. Regardless, there was a clear shift in color variation in the GSE group compared to control, indicating differences in collagen layer quality among groups, which can influence bone mechanical properties [54].

The osteoclast count on the TRAP-stained sections demonstrated a statistically significant increase in the GSE group when compared to GFE. The control group showed high variation. A study in rats evaluated the effect of proanthocyanin on rheumatoid arthritis and its use improved the manifestations of the disease by simultaneously suppressing the differentiation of osteoclasts and promoting the differentiation of osteoblasts [29]. On the contrary, our results show a greater degree of bone remodeling activity. The presence of local inflammation may affect the amount of osteoclasts as reported [55]. However, our results agree with the literature with regard to a trend in increase number of osteoblasts for GSE, although the increase does not seem to be sufficient to counteract the statistically different increase in osteoclast numbers.

It is important to acknowledge that the exact formulations of the over-the-counter GFE and GSE used in this study were not investigated, although it has been well reported that GSE is mostly composed of PAs (there are also other flavonoids such as quercetin, catechin and gallic acid present) [3,56]. For GFE, besides NA/HE, narirutin and other minor components are also present [57]. Nevertheless, it would be difficult to decouple the impact of each of the active compounds within each extract or even their metabolites after ingestion. This study aimed primarily to evaluate the effect of commercially available extracts (thus, mixtures of flavonoids) in oral bone healing and successfully demonstrated that adverse effects in healing are possible.

Future studies that evaluate the influence of tooth site, maxilla vs. mandible, gender, age, dosing and other confounding variables with larger sample sizes are warranted before applying the findings of this pilot study to the general population. In summary, the histological analysis of the GSE group demonstrated a decrease in local bone formation coupled with presence of more immature and less dense bone. Further, increased platelets and fibrous tissue was qualitatively observed at the extraction sites. Our results demonstrate that GSE and GFE affect bone healing parameters during the healing of extraction sockets. However, GFE showed no difference in the amount of bone formation compared to control after 60 days and GSE showed reduced total bone tissue, thus rejecting the hypothesis that both would benefit bone healing. 

In conclusion, dietary supplementation with GFE showed a downregulation of inflammation and mature bone while GSE led to a decrease in bone, collagen density and increased osteoclast numbers in dental extraction sockets. These findings highlight the significance of alternative medicine approaches in clinical outcomes where dietary supplementation with natural compounds can potentially have a major impact on regenerative dentistry.

The present study raises further questions regarding understanding the changes that dietary supplements can elicit in immediate and late alveolar healing and emphasizes the need for continued research on assessing the benefits or harm of using these dietary compounds during oral surgical interventions.

## Supplementary Materials

The following are available online at https://www.mdpi.com/2304-6767/8/3/86/s1, S1 CONSORT checklist; S1 Protocol approved by clinical trial registry.
