# Supplementary material for: Effect of Flavonoid Supplementation on Alveolar Bone Healing—A Randomized Pilot Trial
Source: Dent J (Basel). 2020 Aug 4;8(3):86. doi: 10.3390/dj8030086 (PMC7560062; doi:10.3390/dj8030086)
Supplement: Supplementary file 1 [file dentistry-08-00086-s001.pdf]

CONSORT 2010 checklist of information to include when reporting a randomised trial*

Section/Topic	Item No	Checklist item	Reported on page No
**Title and abstract**			
	1a	Identification as a randomised trial in the title	1
	1b	Structured summary of trial design, methods, results, and conclusions (for specific guidance see CONSORT for abstracts)	2
**Introduction**			
Background and objectives	2a	Scientific background and explanation of rationale	2-4
	2b	Specific objectives or hypotheses	4
**Methods**			
Trial design	3a	Description of trial design (such as parallel, factorial) including allocation ratio	4,5
	3b	Important changes to methods after trial commencement (such as eligibility criteria), with reasons	5
Participants	4a	Eligibility criteria for participants	4
	4b	Settings and locations where the data were collected	4
Interventions	5	The interventions for each group with sufficient details to allow replication, including how and when they were actually administered	4,5
Outcomes	6a	Completely defined pre-specified primary and secondary outcome measures, including how and when they were assessed	N/A
	6b	Any changes to trial outcomes after the trial commenced, with reasons	N/A
Sample size	7a	How sample size was determined	4,5
	7b	When applicable, explanation of any interim analyses and stopping guidelines	N/A
Randomisation:			
Sequence generation	8a	Method used to generate the random allocation sequence	4,5
	8b	Type of randomisation; details of any restriction (such as blocking and block size)	4,5
Allocation concealment mechanism	9	Mechanism used to implement the random allocation sequence (such as sequentially numbered containers), describing any steps taken to conceal the sequence until interventions were assigned	4,5
Implementation	10	Who generated the random allocation sequence, who enrolled participants, and who assigned participants to interventions	4,5
Blinding	11a	If done, who was blinded after assignment to interventions (for example, participants, care providers, those	5

		assessing outcomes) and how	
	11b	If relevant, description of the similarity of interventions	
Statistical methods	12a	Statistical methods used to compare groups for primary and secondary outcomes	9
	12b	Methods for additional analyses, such as subgroup analyses and adjusted analyses	N/A
**Results**			
Participant flow (a diagram is strongly recommended)	13a	For each group, the numbers of participants who were randomly assigned, received intended treatment, and were analysed for the primary outcome	7
	13b	For each group, losses and exclusions after randomisation, together with reasons	N/A
Recruitment	14a	Dates defining the periods of recruitment and follow-up	4
	14b	Why the trial ended or was stopped	N/A
Baseline data	15	A table showing baseline demographic and clinical characteristics for each group	N/A
Numbers analysed	16	For each group, number of participants (denominator) included in each analysis and whether the analysis was by original assigned groups	6
Outcomes and estimation	17a	For each primary and secondary outcome, results for each group, and the estimated effect size and its precision (such as 95% confidence interval)	9-13
	17b	For binary outcomes, presentation of both absolute and relative effect sizes is recommended	N/A
Ancillary analyses	18	Results of any other analyses performed, including subgroup analyses and adjusted analyses, distinguishing pre-specified from exploratory	N/A
Harms	19	All important harms or unintended effects in each group (for specific guidance see CONSORT for harms)	5
**Discussion**			
Limitations	20	Trial limitations, addressing sources of potential bias, imprecision, and, if relevant, multiplicity of analyses	14-16
Generalisability	21	Generalisability (external validity, applicability) of the trial findings	14-16
Interpretation	22	Interpretation consistent with results, balancing benefits and harms, and considering other relevant evidence	14-16
**Other information**			
Registration	23	Registration number and name of trial registry	4
Protocol	24	Where the full trial protocol can be accessed, if available	S1 protocol
Funding	25	Sources of funding and other support (such as supply of drugs), role of funders	1

*We strongly recommend reading this statement in conjunction with the CONSORT 2010 Explanation and Elaboration for important clarifications on all the items. If relevant, we also recommend reading CONSORT extensions for cluster randomised trials, non-inferiority and equivalence trials, non-pharmacological treatments, herbal interventions, and pragmatic trials. Additional extensions are forthcoming: for those and for up to date references relevant to this checklist, see www.consort-statement.org.

PARECER CONSUBSTANCIADO DO CEP

DADOS DO PROJETO DE PESQUISA

Título da Pesquisa: EFEITO DE COMPOSTOS NATURAIS NA REGENERAÇÃO ÓSSEA: ESTUDO EM HUMANOS

Pesquisador: Marco Aurélio Bianchini

Área Temática: Área 8. Pesquisa com cooperação estrangeira.

Versão: 2

CAAE: 09884612.4.0000.0121

Instituição Proponente: UNIVERSIDADE FEDERAL DE SANTA CATARINA

Patrocinador Principal: Financiamento Próprio

DADOS DO PARECER

Número do Parecer: 208.999

Data da Relatoria: 18/02/2013

Apresentação do Projeto:

Serão selecionados 20 pacientes adultos do sexo masculino, que necessitam da extração do elemento dental e instalação de implantes orais, de modo a possibilitar a reabilitação protética. Os pacientes selecionados para o estudo serão distribuídos em quatro (04) grupos de acordo com o tipo de fitoquímico utilizado: GRUPO 1: Extrato de semente de uva [PA]; GRUPO 2: Extrato de Gardênia [GE]; GRUPO 3: Extrato de Toranja [NA / HE] e; GRUPO 4: Grupo controle (nenhum fitoquímico/tratamento, NTx). Cada tratamento [PA, GE, NA / HE, e grupo controle (NTx)] será realizado em 10 alvéolos pós-extração. Cada paciente deverá ingerir 125 MG do fitoquimico especifico 3 vezes ao dia com refeições (extrato de semente de uva [PA], extrato de gardênia [GE], e extrato de toranja [NA/HE]), duas semanas antes da cirurgia e manter esse regime durante oito semanas após a extração (10 semanas totais) até o momento da instalação do implante. Amostras serão coletadas no interior do alvéolo ¿ no dia da extração (amostra 1) e após 60 dias da extração (amostra 2)

Objetivo da Pesquisa:

Objetivo Primário: Determinar se a exposição sistêmica contínua aos compostos, durante a cicatrização do sítio de extração, leva à formação óssea de alta qualidade. Objetivo Secundário: Avaliar os efeitos da ingestão de PA (semente de uva), GE (extrato de gardênia) e HE/NA (extrato de toranja) na expressão genética dos fatores de crescimento.

Avaliação dos Riscos e Benefícios:

Sempre há o risto, ainda que não intencional e inesperado, de quebra de sigilo.

Comentários e Considerações sobre a Pesquisa:

Sem comentários adicionais.

Considerações sobre os Termos de apresentação obrigatória:

A folha de rosto vem assinada pelo pesquisador responsável e pelo chefe do Departamento de Odontologia, que assina também uma declaração de que tomou conhecimento da pesquisa e que cumprirá os termos da Res. 196/96 e complementares. Também é apresentada declaração semelhante do chefe do Departamento de Periodontia da Universidade da Pennsylvania. A declaração traz o timbre da instituição e está em português. O TCLE é longo e com muitos termos técnicos, mas esclarece os objetivos da pesquisa e os procedimentos que serão realizaods.

Recomendações:

Sem recomendações adicionais.

Conclusões ou Pendências e Lista de Inadequações:

Sem pendências.

Situação do Parecer:

Aprovado

Necessita Apreciação da CONEP:

Sim

Considerações Finais a critério do CEP:

Por inadequação do sistema da Plataforma Brasil, não há, salvo melhor juizo, maneira de não encaminhar à CONEP o presente projeto.

O presente projeto, seguiu nesta data para análise da CONEP e só tem o seu início autorizado após a aprovação pela mesma.

FLORIANOPOLIS, 01 de Março de 2013

Assinador por:
Washington Portela de Souza
(Coordenador)

Project presentation:

Adult male patients will be selected (up to 20 patients per group) that are in need of extraction of a tooth with subsequent plan for a dental implant. Qualified patients will be distributed in 4 groups: group 1 GSE – grape seed extract, group 2 GE – gardenia extract, group 3 GFE – grape fruit extract, and group 4 CON – control/no treatment. Each treatment will be analyzed at a minimum in 10 dental extractions. Each patient will ingest at least 125mg of the phytochemical 3x a day 2 weeks prior to the extraction and will continue for 8 weeks after extraction. Samples will be collected from the healing alveolar bone after 1 and 60 days post-extraction.

Objective of the research:

To determine if systemic exposure to natural compounds during the healing of the dental extraction site will lead to changes in bone healing and bone quality including gene expression of factors important in bone such as growth factors.

PARECER CONSUBSTANCIADO DA CONEP

DADOS DO PROJETO DE PESQUISA

Título da Pesquisa: EFEITO DE COMPOSTOS NATURAIS NA REGENERAÇÃO ÓSSEA: ESTUDO EM HUMANOS

Pesquisador: Marco Aurélio Bianchini

Área Temática: Genética Humana:
(Haverá envio para o exterior de material genético ou qualquer material biológico humano para obtenção de material genético, salvo nos casos em que houver cooperação com o Governo Brasileiro;);
Pesquisas com coordenação e/ou patrocínio originados fora do Brasil, excetuadas aquelas com copatrocínio do Governo Brasileiro;

Versão: 4

CAAE: 09884612.4.0000.0121

Instituição Proponente: UNIVERSIDADE FEDERAL DE SANTA CATARINA

Patrocinador Principal: Financiamento Próprio

DADOS DO PARECER

Número do Parecer: 740.532

Data da Relatoria: 28/07/2014

Apresentação do Projeto:

INTRODUÇÃO

"Vários compostos que estão presentes em plantas e alimentos, têm sido indicados para uma grande variedade de problemas de saúde como inflamação, bacteremia e câncer 3-6. Muitos compostos naturais são conhecidos por terem efeitos positivos sobre a resistência do colágeno, massa de tecido e proliferação das células. Desses compostos, os três têm demonstrado potencial para reduzir a digestão/degradação do colágeno: extrato de semente de uva (contendo proantocianidinas, PA), extrato da planta gardênia (contendo genipin, GE) e extrato de toranja (contendo hesperidina, HE e naringenina, NA). No entanto, seus efeitos sobre a organização de ligações cruzadas na matriz, bem como a função nas células não foram claramente investigados. Com base nos efeitos positivos relatados acima, sugere-se que os efeitos dos primeiros mecanismos moleculares sobre a expressão do fator de crescimento e da qualidade de matriz de colágeno podem afetar a formação óssea significativamente, em longo prazo. Enquanto há apoio para se recomendar dietas ricas em nutrientes provenientes de frutas e legumes, são limitadas as

evidências de que fitoquímicos possam ter algum efeito fisiológico significante. Isto se justifica, especialmente, pela baixa biodisponibilidade após ingestão. Há uma escassez de pesquisas que demonstrem como a biodisponibilidade aumentada (através da suplementação) poderá afetar o corpo humano e, também os mecanismos de ação. Deste modo, pesquisas que objetivam avaliar as atividades biológicas de fitoquímicos se justificam. Entre os suplementos fitoquímicos mais consumidos são os compostos fenólicos (a partir de frutas e vegetais) e as terpins (de plantas). Compostos fenólicos ou flavonoides são sub-classificados como antocianidinas, flavonois, flavanonas, flavonoides, flavonas e isoflavonas. Um dos flavonoides mais populares disponíveis comercialmente é o de extrato de semente de uva, o qual é uma mistura de flavonoides chamados proantocianidinas (PA) em forma de monômeros e polímeros (também em maçãs, uvas, vinho tinto e chocolate). O extrato de semente de uva tem sido amplamente utilizado com a promessa de ser antimicrobiano, prevenir a inflamação e proteger o sistema cardiovascular. Recentemente, observou-se que o PA é um agente de ligações cruzadas do colágeno tipo I, que não impede, porém a colonização bacteriana da dentina. Outro composto fenólico disponível comercialmente é a flavanona (presente em frutos cítricos). HE e NA, ambas as flavanonas no extrato de toranja, foram estudados em células. HE foi demonstrada estimular a diferenciação dos osteoblastos por meio de ativação das funções da proteína morfogenética óssea (BMP), enquanto que NA foi demonstrada melhorar a massa óssea em ratos por meio de injeções intra-gástricas. O outro grupo popular de fitoquímicos utilizado em aplicações na saúde é de terpins. Neste grupo, a genipin (GE), que é derivada da fruta Gardênia jasminoides Ellis, é considerada o composto mais extensamente estudado. A investigação neste composto tem-se concentrado na sua capacidade de gerar ligações cruzadas em materiais de enxerto, além do seu extenso uso no tratamento da hipertensão e prevenção da diabetes. Por tudo isso, PA, GE e HE/NA poderiam ser utilizados em condições fisiológicas e/ou patológicas (na melhora da resistência óssea mecânica e a degradação o enzimática por meio da ligação cruzada do colágeno; e na melhora das funções celulares através da disponibilidade de fatores de crescimento) e supostamente levariam a uma melhor função e consequentemente qualidade de vida. No campo de Periodontologia e Implantologia, fitoquímicos podem resultar em melhoria da remodelação óssea, redução do progresso da doença periodontal por meio do amadurecimento da matriz colágena, regular secreção de fatores de crescimento, e muitas outras funções ainda não reveladas. Diante disso o objetivo do projeto proposto é avaliar os efeitos da ingestão de PA, GE e HE/NA na expressão genética dos fatores de crescimento. Ainda determinar se a exposição sistêmica contínua aos compostos, durante a cicatrização do sítio de extração, leva à formação óssea de alta qualidade. Após aprovação do projeto pelo Comitê de Ética

em Pesquisa com Seres Humanos, localizado na Universidade Federal de Santa Catarina, e do Intitutional Review Board, localizado na Universidade da Pennsylvania, EUA, serão selecionados pacientes adultos do sexo masculino, que necessitam da extração do elemento dental e instalação de implantes orais, de modo a possibilitar a reabilitação protética. Os pacientes selecionados para o estudo serão distribuídos em quatro (04) grupos de acordo com o tipo de fitoquímico utilizado: GRUPO 1: Extrato de semente de uva [PA]; GRUPO 2: Extrato de Gardênia [GE]; GRUPO 3: Extrato de Toranja [NA / HE] e; GRUPO 4: Grupo controle (nenhum fitoquímico/tratamento, NTx) Cada tratamento [PA, GE, NA / HE, e grupo controle (NTx)] será realizado em 10 alvéolos pós-extração. Cada paciente deverá ingerir 125 MG do fitoquimico especifico 3 vezes ao dia com refeições (extrato de semente de uva [PA], extrato de gardênia [GE], e extrato de toranja [NA/HE], Vitamin Shoppe, NJ, EUA), duas semanas antes da cirurgia e manter esse regime durante oito semanas após a extração (10 semanas totais) até o momento da instalação do implante. Amostras serão coletadas no interior do alvéolo - no dia da extração (amostra 1) e após 60 dias da extração (amostra 2). AMOSTRA 1 O espécime de biópsia será imediatamente colocado em RNAlater (Qiagen, CA, EUA), para o transporte evitando a degradação do material. O RNA será extraído com o reagente Trizol (Invitrogen, California, EUA) e 2 ¿g de RNA total, irá ser convertido em cDNA usando o kit da transcriptase reversa Omniscript kit (Qiagen). PCR quantitativo (qRT-PCR) será realizado utilizando os iniciadores de sequência específica para os fatores de crescimento de BMP-4 (Hs01041266_m1, Applied Biosystems, CA, EUA), BMP-7 (Hs00233476_m1, Applied Biosystems), o TGF-beta (Hs00998133_m1, Applied Biosystems) e PDGF (Hs00964426_m1, Applied Biosystems). As reações serão preparadas e analisadas em triplicado por Applied Biosystems StepOnePlusTM (Applied Biosystems). O nível de expressão de mRNA dos genes de interesse serão calculados em relação à gliceraldeído-3-desidrogenase (GAPDH) (Hs02758991_g1, Applied Biosystems) e os dados serão analisados pelo 2^delta-delta método CT 21. Os participantes serão acompanhados durante todo o período de cicatrização. AMOSTRA 2 Cada espécime será colocado em formalina tamponada a 10% durante três dias e depois desmineralizado com 0,5 M EDTA pH 7,4, durante quatro semanas. As amostras serão desidratadas em várias gradações ascendentes de etanol (50-100%), embebido em xilol e embebidos em parafina. Secções de 6 microm de cada bloco de parafina serão cortadas utilizando um micrótomo Leica RM204 Jung (Leica, Alemanha). As secções serão manchadas pelo tricrômico de Masson para avaliação histomorfométrica e por Picrosirius Red (PSR) para avaliar a organização do colágeno e maturação. A quantificação de cores da PSR será feito pelo software Image J (NIH, MA, EUA. Dados de PCR serão quantificados por One-Way ANOVA e pelo teste de Kruskall Wallis PSR (JMP, SAS, NC, EUA). A compreensão de como

agem esses agentes naturais poderia abrir caminho para desenvolvê-las em terapias para remodelação do tecido em periodontia e outras áreas clínicas."

HIPÓTESE:
"Compostos naturais ou fitoquímicos têm sido amplamente utilizados no tratamento de diversas patologias desde a bacteremia até o câncer. In vitro, observa-se que alguns compostos afetam a proliferação de células, aumentam a massa de tecido e promovem a ligação cruzada do colágeno no tecido conjuntivo. Os mecanismos por trás dessas funções ainda não foram descobertos e as pesquisas estão apenas nos seus passos iniciais. Diante de tais efeitos positivos observados in vitro e a disponibilidade de tais compostos para o consumo humano, o efeito de doses fisiológicas em células e matriz tecidual in vivo devem ser exploradas. Com base nos dados atuais, pode-se supor que certos compostos naturais afetam osso não só pela sua capacidade não enzimática de criar ligações cruzadas no colágeno, mas também pela capacidade de alteração das respostas celulares como mudanças na disponibilidade de fatores de crescimento. Este estudo piloto irá abordar os seguintes objetivos específicos: 1. Seres humanos irão ingerir compostos naturais diariamente antes da extração dental até a colocação do implante dentário. Através de biópsias de tecido conjuntivo alveolar/osso, alguns fatores de crescimento importantes para a manutenção e reparação, ou seja, proteína morfogenética óssea (BMP) 4 e 7, fator de transformação do crescimento (TGF)-beta, e fator de crescimento derivado de plaquetas (PDGF) irá ser testada por meio de expressão dos genes 24 hs após extração dentária. 2. Qualidade óssea será acessada antes da colocação do implante no dia 60, por meio de análises histológicas de colágeno. A compreensão de como agem esses agentes naturais poderia abrir caminho para desenvolvê-las em terapias para remodelação do tecido em periodontia e outras áreas clínicas."

METODOLOGIA:
"Após aprovação do projeto pelo Comitê de Ética em Pesquisa com Seres Humanos, localizado na Universidade Federal de Santa Catarina, e do Intitutional Review Board, localizado na Universidade da Pennsylvania, EUA. Os pacientes serão triados na clínica odontológica do Centro de Estudos em Implantes Dentários "CEPID" situada na Universidade Federal de Santa Catarina. Todos os pacientes submetidos à pesquisa assinarão um Termo de Consentimento Livre e Esclarecido autorizando sua participação e coleta dos dados clínicos e histológicos.Serão formados quatro (04) grupos de acordo com o tipo de fitoquímico utilizado: GRUPO 1: Extrato de semente de uva [PA], GRUPO 2: para vermelho representa matriz compacta, madura e organizada 22. Dados de PCR serão

quantificados por One -Way ANOVA e pelo teste de Kruskall Wallis PSR (JMP, SAS, NC, EUA)."

Critérios de Inclusão: "Serão selecionados pacientes adultos do sexo masculino, que necessitam da extração do elemento dental e instalação de implantes orais, de modo a possibilitar a reabilitação protética implantossuportada."

Critérios de Exclusão: "Serão excluídos os pacientes que apresentem histórico de quimioterapia ou radioterapia de cabeça e/ou no pescoço 24 meses antes da primeira consulta, doença periodontal prévia, pacientes etílicos, história de terapia de bisfosfanatos, diabéticos descompensados, pacientes fumantes e/ou que apresentem qualquer contraindicação para cirurgia oral.".

Objetivo da Pesquisa:
Objetivo Primário:
"Determinar se a exposição sistêmica contínua aos compostos, durante a cicatrização do sítio de extração, leva à formação óssea de alta qualidade.".

Objetivos Secundários:
"Avaliar os efeitos da ingestão de PA, GE e HE/NA na expressão genética dos fatores de crescimento."

Avaliação dos Riscos e Benefícios:
"Os riscos da pesquisa ficam relacionados aos riscos operatórios das etapas 1 e 2. Os principais riscos operatórios inerentes e potenciais ao procedimento incluem, mas não são limitados a eles, os seguintes:
1.Desconforto pós-operatório e edema que pode necessitar de alguns dias de recuperação. 2. Sangramento continuado que pode ser prolongado.
3. Infecção pós-operatória que pode exigir tratamento adicional.
4.Restrição da abertura bucal por alguns dias ou semanas.
5. Estiramento da abertura bucal por alguns dias
ou semanas.
6. Injúria ao nervo subjacente aos dentes, resultando em adormecimento ou formigamento do lábio, queixo, bochechas, gengiva, dentes e/ou língua do lado operado,que pode persistir por semanas,meses ou, em raras circunstâncias, permanente.
7. Comunicação com o seio maxilar (uma cavidade normal localizada acima

dos dentes superiores), exigindo cirurgia adicional.
8. Rejeição a materiais implantados implantes,membranas, materiais sintéticos).
9. Deiscência de sutura expondo a área operada.
10. Necessidades de reintervenção cirúrgicas ou de um novo planejamento.Como todos os pacientes recrutados nesse estudo receberão implantes orais, pode-se considerar que todos
sejam beneficiados pela melhora da qualidade óssea - pela ingestão dos fitoquímicos. Ainda não haverá nenhum custo referente aos procedimentos de preservação alveolar (membranas e materiais para regeneração óssea)."

Comentários e Considerações sobre a Pesquisa:

Trata-se de um recurso, com o objetivo de responder às pendências não atendidas ou parcialmente atendidas, sendo elas: 2,6A, 6B, 6C, 6E, 6F, 6I, 6K e 8.

Considerações sobre os Termos de apresentação obrigatória:

Verificar item "Conclusões ou Pendências e Lista de Inadequações".

Recomendações:

Verificar item "Conclusões ou Pendências e Lista de Inadequações".

Conclusões ou Pendências e Lista de Inadequações:

RECURSO AO PARECER CONSUBSTANCIADO CONEP N° 670993:

1. Como as análises laboratoriais serão desenvolvidas na Universidade da Pensilvânia e, no caso serão feitas análises de expressão gênica, deve ser incluída a Área Temática Especial de "Genética Humana", na qual haverá envio e armazenamento de material genético ao exterior. Solicita-se adequação.
RESPOSTA: Corrigido.
ANÁLISE: PENDÊNCIA ATENDIDA.

2. Solicita-se que seja apresentado orçamento financeiro detalhado, que especifique todos os recursos, fontes e destinação, em especial os custos operacionais (recursos humanos e materiais), bem como qual é a fonte financiadora.
RESPOSTA: Materiais de Consumo (Orçamento). Vide documento "NOVO. EFEITO DE COMPOSTOS NATURAIS NA REGENERAÇÃO ÓSSEA ESTUDO EM HUMANOS.doc"
ANÁLISE: PENDÊNCIA NÃO ATENDIDA. No orçamento apresentado, não consta a previsão de

ressarcimento aos participantes da pesquisa. Ademais, nada é informado sobre o custo do implante, e em especial sobre a reposição do implante perdido (caso ocorra), durante a fase de osteointegração, visto que a membrana utilizada não é efetiva pra impedir a invasão de tecido.

RECURSO:
• O valor estimado de ressarcimento dos participantes da pesquisa foi adicionado ao orçamento
• Quanto aos custos dos implantes e reposição, em caso de perda, informo que será de responsabilidade do Centro de Ensino e Pesquisas em Implantes dentários, localizado na Universidade Federal de Santa Catarina conforme o TCLE que foi corrigido... “Não será cobrado nenhum valor referente aos procedimentos da pesquisa. Após a realização da pesquisa o(s) implante(s) será (ao) imediatamente instalado (s) pelos alunos do curso de Especialização de Implantodontia CEPID-UFSC e você deverá arcar com as despesas dos implantes e reabilitação protética, repassadas pelo CEPID-UFSC”.
ANÁLISE DO RECURSO: • No arquivo “EFEITO DE COMPOSTOS NATURAIS NA REGENERAÇÃO ÓSSEA. RECURSO.docx” consta o valor estimado para despesas com pacientes. Uma vez que a pesquisa objetiva a) avaliar os efeitos da ingestão de PA, GE e HE/NA na expressão genética dos fatores de crescimento. e b) determinar se a exposição sistêmica contínua aos compostos, durante a cicatrização do sítio de extração, leva à formação óssea de alta qualidade e para isso os pesquisadores irão recrutar participantes que necessitam de exodontia e posterior instalação de implantes, a instalação destes não são de responsabilidade dos pesquisadores. RECURSO ATENDIDO.

3. Quanto ao cronograma apresentado, o mesmo não está adequado, pois informa que o estudo já teve início. Solicita-se esclarecimento e, caso necessário, adequação do cronograma com relação à data de início do estudo, dado que o mesmo ainda se encontra em análise no sistema CONEP/CEP até a presente data.
RESPOSTA: CRONOGRAMA. Vide documento "NOVO. EFEITO DE COMPOSTOS NATURAIS NA REGENERAÇÃO ÓSSEA ESTUDO EM HUMANOS.doc"
ANÁLISE: PENDÊNCIA ATENDIDA.

4. Não está claro no Protocolo se o procedimento de colocação do implante será realizado imediatamente após a biópsia do dia 60. Solicita-se esclarecimento.
RESPOSTA:...4.3.2 Etapa II: No dia 60 da extração do elemento dental, sob anestesia, um retalho de

espessura total será levantado e um espécime cilíndrico de 3 mm será obtido pelo uso de uma trefina, antes da colocação do implante. Imediatamente após a biópsia, os implantes serão instalados conforme o protocolo estabelecido no item cirurgia II desse projeto, sem nenhum risco adicional ao paciente.
ANÁLISE: PENDÊNCIA ATENDIDA.

5. Os procedimentos experimentais implicam em riscos. A biópsia a se realizar 24 horas após a exodontia e a do dia 60 trarão riscos? Quais? Solicita-se esclarecimentos.
RESPOSTA: ...4.3.1 Etapa I: Após 24 horas da extração dos dentes, sob anestesia local, a membrana collatape será perfurada para acessar o tecido subjacente. Posteriormente, uma pequena quantidade de tecido mole localizada no interior do alvéolo será removida com auxílio de perfurador de tecido de 4mm (Salvin, NC, EUA). O local será recoberto com Collaplug (Cimmer, CA, EUA), após a remoção da amostra. Tal conduta poderá gerar pequeno desconforto pós-operatório, contudo o paciente já estará sob efeito de medicação analgésica. .... 4.3.2 etapa II: No dia 60 da extração do elemento dental, sob anestesia, um retalho de espessura total será levantado e um espécime cilíndrico de 3 mm será obtido pelo uso de uma trefina, antes da colocação do implante. Imediatamente após a biópsia, os implantes serão instalados conforme o protocolo estabelecido no item cirurgia II desse projeto, sem nenhum risco adicional ao paciente....
ANÁLISE: PENDÊNCIA ATENDIDA.

6. Quanto ao Termo de Consentimento Livre e Esclarecido:

a) Os três primeiros parágrafos estão em forma de declaração, ao invés de estar sendo o participante da pesquisa convidado a participar do estudo. Essa forma de apresentação não pode ser aceita. O consentimento deve ser solicitado em forma de convite, esclarecendo em linguagem acessível todos os procedimentos e garantias (Resolução CNS 466/12, item IV) para, ao final, o participante declarar que está de acordo. Deve-se garantir que o participante da pesquisa possui o direito de retirar o consentimento sem sofrer qualquer tipo de penalização. É inadequado começar com "por mim assinado, autorizo...". Esses parágrafos devem finalizar o TCLE. No corpo do documento as frases "dou pleno consentimento...", "Fui informado..." devem ser substituídas por afirmações do pesquisador e não do participante da pesquisa. Esse procedimento pode confundir o participante e comprometer sua autonomia. Solicita-se adequação.
RESPOSTA: Você está sendo convidado para participar, como voluntário, em uma pesquisa. Após

ser esclarecido sobre as informações a seguir, no caso de aceitar fazer parte do estudo, assine ao final deste documento, que está em duas vias. Uma delas é sua e a outra é do pesquisador responsável. Desde logo fica garantido o sigilo das informações. Em caso de recusa você não será penalizado de forma alguma. Informações sobre a pesquisa:
ANÁLISE: PENDÊNCIA PARCIALMENTE ATENDIDA. Ainda no TCLE podem ser identificados trechos onde foi utilizada a primeira pessoa. Como exemplo é possível citar: "Se qualquer condição não prevista acima ocorrer durante a cirurgia, confio..."; "Entendo que serei ressarcido de todas as despesas decorrentes da participação..."; "Estou ciente de que medicamentos, drogas, e anestésicos prescritos podem causar sonolência..."; "...Portanto, fui aconselhado(a) a não trabalhar..."; "Entendo que durante a pesquisa, será cobrado apenas o valor dos implantes..."; "... Estou ciente que após a segunda etapa, serei encaminhado ao curso..."; "Compreendo que os tratamentos

RECURSO:
Este item foi corrigido no TCLE....
“Se qualquer condição não prevista acima ocorrer durante a cirurgia, você deverá confiar no julgamento do aluno/professor/assistente – Marco Aurélio Bianchini e João Gustavo Oliveira de Souza – para realizar procedimentos adicionais (intervenções cirúrgicas, materiais, prescrição de medicação, tratamento de infecção) sem gastos adicionais.
Você será ressarcido de todas as despesas decorrentes da participação no estudo, tais como transporte e alimentação nos dias em que for necessária sua presença para consultas ou exames.
Você de verá estar ciente de que medicamentos, drogas e anestésicos prescritos podem causar sonolência e dificuldade de atenção ou coordenação, e que alergias podem ser aumentadas com o uso concomitante de bebidas alcoólicas ou outros medicamentos. Portanto você será aconselhado (a) a não trabalhar e nem operar qualquer veículo ou dispositivos perigosos enquanto estiver sob vigência de medicamentos e /ou drogas até estar completamente recuperado do efeito dos mesmos, por, no mínimo, 24 horas após ter sido liberado da cirurgia e recuperado dos efeitos da medicação anestésica que me foi dada.
Não será cobrado nenhum valor referente aos procedimentos da pesquisa. Após a realização da pesquisa o(s) implante(s) será (ao) imediatamente instalado (s) pelos alunos do curso de Especialização de Implantodontia CEPID-UFSC e você deverá arcar com as despesas dos implantes e reabilitação protética, repassadas pelo CEPID-UFSC.
Você teve a oportunidade de ler e entender completamente os termos e palavras contidas no texto

Endereço: SEPN 510 NORTE, BLOCO A 1º SUBSOLO, Edifício Ex-INAN - Unidade II - Ministério da Saúde
Bairro: Asa Norte **CEP:** 70.750-521
UF: DF **Município:** BRASILIA
Telefone: (61)3315-5878 **E-mail:** conep@saude.gov.br

acima e foram dadas explicações referentes a todos os itens ou declarações acima. Você declara que sabe ler e escrever em português
É importante salientar que a pesquisa tem o objetivo de avaliar tanto os efeitos da ingestão de PA, GE e HE/NA na expressão genética dos fatores de crescimento, quanto determinar se a exposição sistêmica contínua aos compostos, durante a cicatrização do sítio de extração, leva à formação óssea de alta qualidade. Nesse contexto informamos que a etapa de instalação dos implantes e confecção da prótese são etapas que não correspondem à pesquisa..... Estas etapas serão realizadas no mesmo local (Centro de Ensino e Pesquisa em Implantes Dentários, UFSC) pelo curso de Especialização em Implantodontia.
ANÁLISE: RECURSO ATENDIDO.

b) Cabe lembrar que o TCLE é o documento onde o pesquisador deve prestar todas as informações pertinentes ao protocolo para os participantes da pesquisa de maneira clara e objetiva. Além disso, por utilizar linguagem técnica, fora do alcance da população comum, não cabe indicar os procedimentos técnicos envolvidos, razão pela qual solicita-se adequação do documento como um todo
RESPOSTA: Todo o TCLE foi corrigido com linguagem adequada.
ANÁLISE: PENDÊNCIA NÃO ATENDIDA. No TCLE ainda constam termos técnicos como, por exemplo: expressão genética dos fatores de crescimento; exposição sistêmica; sítio de extração; longevidade da reabilitação implantossuportada; MEMBRANA será posicionada de modo a recobrir o ALVÉOLO; pequena quantidade de tecido mole; interior do alvéolo; biossegurança; protocolo anestésico; riscos operatórios inerentes; procedimentos cirúrgicos; pós-operatório; edema; infecção; Estiramento da abertura bucal; INJÚRIA ao nervo SUBJACENTE aos dentes; Deiscência de sutura; osseointegração; reintervenção cirúrgicas; intervenções cirúrgicas; prescrição de medicação; etapa protética.
RECURSO:
Este item foi corrigido no TCLE. “O objetivo do estudo proposto é avaliar os efeitos da ingestão de fitoquímicos (extrato de semente de uva [PA], extrato de gardênia [GE], e extrato de toranja [NA/HE], Vitamin Shoppe, NJ, EUA) nos fatores que estão relacionados ao aumento de quantidade e qualidade óssea. Ainda determinar se a exposição sistêmica contínua aos compostos, durante a cicatrização do local da extração, leva à formação óssea de alta qualidade. Essas “melhoras” supostamente levariam a uma melhor função dos implantes e consequentemente maior longevidade dos implantes e respectivas próteses. Os procedimentos necessários para pesquisa

serão executados na seguinte sequência:

Etapa I:
Os elementos dentais condenados serão extraídos e uma película (membrana) será posicionada de modo a recobrir o local da extração. Após 24 horas da extração dos dentes, uma pequena quantidade de gengiva localizada no interior do local da extração será removida, sendo que o local será recoberto, após a remoção da amostra.

Etapa/Cirurgia II
No dia 60 após a extração do elemento dental, uma pequena amostra uma pequena quantidade de gengiva localizada no interior do local da extração será removida, e o implante será imediatamente instalado pelos alunos do Curso de Especialização em Implantodontia, no mesmo local onde foi removida a amostra, não oferecendo nenhum prejuízo ou dano adicional ao paciente.
Todas as etapas serão realizadas em ambiente limpo, com anestesia e medicamentos adequados.
Os principais riscos dos procedimentos cirúrgicos incluem, mas não são limitados a eles, os seguintes:
1.Desconforto após a cirurgia e “inchaço” que pode necessitar de alguns dias de recuperação
2.Sangramento continuado que pode ser prolongado.
3.Contaminação após a cirurgia que pode exigir tratamento adicional.
4.Diminuição da abertura bucal por alguns dias ou semanas.
5.Pequeno corte no canto da boca por alguns dias ou semanas.
6.Fratura de mandíbula.
7.Trauma ao nervo próximo dos dentes, resultando em adormecimento ou formigamento do lábio, queixo, bochechas, gengiva, dentes e/ou língua do lado operado, que pode persistir por semanas, meses ou, em raras circunstâncias, permanente.
8.Comunicação com o seio maxilar (uma cavidade normal localizada acima dos dentes superiores), exigindo cirurgia adicional.
9.Rejeição a materiais (membranas, materiais sintéticos)
10.Afrouxamento da sutura “pontos” expondo a área operada.
11.Necessidades de outras cirurgias ou de um novo planejamento.
Os riscos dos procedimentos da coleta das amostras incluem apenas um aumento do desconforto após a cirurgia, no momento da coleta 24 horas. Contudo você já estará sob efeito de medicação analgésica.

ANÁLISE: RECURSO ATENDIDO.

c) Não é informado que está garantido o ressarcimento de gastos relacionados ao estudo. Cabe ressaltar que, como prevê o item IV.3.g da Resolução CNS 466/12, deve ser garantido ao participante da pesquisa o ressarcimento de despesas decorrentes da participação no estudo, tais como transporte e alimentação nos dias em que for necessária sua presença para consultas ou exames. Assim sendo, solicita-se que a garantia de ressarcimento dos gastos decorrentes da participação no estudo seja apresentada de modo claro e afirmativo.
RESPOSTA:...Entendo que serei ressarcido de todas as despesas decorrentes da participação no estudo, tais como transporte e alimentação nos dias em que for necessária sua presença para consultas ou exames...
ANÁLISE: PENDÊNCIA PARCIALMENTE ATENDIDA. A garantia de ressarcimento foi dada, mas deve ser descrita na terceira pessoa.

RECURSO:
Este item foi corrigido no TCLE: “Você será ressarcido de todas as despesas decorrentes da participação no estudo, tais como transporte e alimentação nos dias em que for necessária sua presença para consultas ou exames”
ANÁLISE: RECURSO ATENDIDO.

d) Não há menção ou restrição de participação de pessoas grávidas ou que venham a engravidar durante a referida pesquisa. Solicita-se adequação.
RESPOSTA:...Os pacientes selecionados para este estudo deverão ser do sexo masculino e apresentar dentes que necessitam da extração e posterior instalação de implantes orais, de modo a possibilitar a reabilitação protética implantossuportada...
ANÁLISE: PENDÊNCIA ATENDIDA.

e) Adicionalmente, não consta no TCLE quais seriam as medidas adotadas pelo pesquisador responsável e pela instituição, quanto aos casos de eventos adversos e possíveis danos/lesões aos participantes da pesquisa. Solicita-se adequação.
RESPOSTA:...Se qualquer condição não prevista acima ocorrer durante a cirurgia, confio no julgamento do aluno/professor/assistente – Marco Aurélio Bianchini e João Gustavo Oliveira de

Souza – para realizar procedimentos adicionais (intervenções cirúrgicas, materiais, prescrição de medicação, tratamento de infecção) sem gastos adicionais...
ANÁLISE: PENDÊNCIA PARCIALMENTE ATENDIDA. Embora os pesquisadores tenham descrito as medidas que serão adotadas em casos de intercorrências, o texto deve ser escrito na terceira pessoa.
RECURSO:
Este item foi corrigido no TCLE:
Se qualquer condição não prevista acima ocorrer durante a cirurgia, você deverá confiar no julgamento do aluno/professor/assistente – Marco Aurélio Bianchini e João Gustavo Oliveira de Souza – para realizar procedimentos adicionais (outras cirurgias, materiais, prescrição de medicação, tratamento de infecção "contaminação") sem gastos adicionais.
ANÁLISE: RECURSO ATENDIDO.

f) Considerando que o participante da pesquisa tem direito ao sigilo e à confidencialidade e a equipe de pesquisa tem o dever de garanti-los, deve constar no TCLE de modo claro e afirmativo que será assegurada a garantia de sigilo, de acordo com as normas brasileiras, com a descrição dos procedimentos para tal, ou seja, como os dados serão anonimizados antes de serem encaminhados pela equipe médica responsável pelos cuidados do participante do estudo para qualquer outra instância, sejam outros pesquisadores ou o patrocinador, se for o caso. Solicita-se adequação
RESPOSTA: ...A pesquisa será realizada no centro de estudos e pesquisas em implantes dentários – CEPID; no centro cirúrgico da Universidade Federal de Santa Catarina – CCS – UFSC; e as análises laboratoriais serão realizadas no Intitutional Review Board, localizado na universidade da Pennsylvania, EUA. ...Certifico que tive oportunidade de ler e entender completamente os termos e palavras contidas no texto acima e me foram dadas explicações referentes a ele e que todos os itens ou declarações foram preenchidos no momento. Também declaro que sei ler e escrever português. Eu, ___________________________________, abaixo assinado, fui informado e esclarecido pelo pesquisador ___________________________ sobre a pesquisa, os procedimentos nela envolvidos, assim como os possíveis riscos e benefícios decorrentes de minha participação. Foi-me garantido o sigilo das informações e que posso retirar meu consentimento a qualquer momento, sem que isto leve a qualquer penalidade ou interrupção de meu acompanhamento/assistência/tratamento. Local e data _______________/_______/_______/__________/...Não haverão outros pesquisadores ou patrocinadores envolvidos no trabalho.

ANALISE: PENDÊNCIA NÃO ATENDIDA. Não está claro no TCLE como os dados obtidos serão anonimizados antes de serem enviados à Universidade da Pennsylvania - EUA.

RECURSO:
Este item foi corrigido no TCLE:
Antes de serem enviados à Universidade da Pennsylvania – EUA seus dados receberão um código com as inicias do primeiro e último nome a fim de preservar sua identidade
ANÁLISE: RECURSO ATENDIDO.

g) Deve ser informado que o mesmo será elaborado em duas vias, sendo uma retida com o pesquisador responsável e outra com o participante da pesquisa (Resolução CNS 466/12 itens IV.3.f e IV.5.d). Solicita-se adequação.
RESPOSTA: Você está sendo convidado para participar, como voluntário, em uma pesquisa. Após ser esclarecido sobre as informações a seguir, no caso de aceitar fazer parte do estudo, assine ao final deste documento, que está em duas vias. Uma delas é sua e a outra é do pesquisador responsável. Desde logo fica garantido o sigilo das informações. Em caso de recusa você não será penalizado de forma alguma. Informações sobre a pesquisa:
ANÁLISE: PENDÊNCIA ATENDIDA.

h) Não foi apresentada nenhuma forma de contato com o CEP responsável pelo acompanhamento do estudo ou com o pesquisador responsável. Solicita-se que seja incluso no TCLE uma breve descrição do que é o CEP, qual sua função no estudo, seu endereço, horário de funcionamento e as suas formas de contato. Solicita-se adequação.
RESPOSTA:Título: EFEITO DE COMPOSTOS NATURAIS NA REGENERAÇÃO ÓSSEA: ESTUDO EM HUMANOS. Pesquisador Responsável: Marco Aurélio Bianchini Telefone para contato: (48) 37219077. email: bian07@yahoo.com.br Pesquisador Participante: João Gustavo Oliveira de Souza telefones para contato: (48) 37219077 / (48) 96590009. Email: joaogustavo_s@hotmail.com Comitê de Ética em Pesquisas com Seres Humanos (CEPSH): Biblioteca Universitária Central -Setor de Periódicos (térreo). Telefone: (48) 37219206. Horário de atendimento: das 10:00 hs – 12:00 hs e 14:00 hs – 16:00 hs de segunda a sexta-feira. O CEPSH é um órgão colegiado interdisciplinar, deliberativo, consultivo e educativo, vinculado à Universidade Federal de Santa Catarina, mas independente na tomada de decisões, criado para defender os interesses dos sujeitos da pesquisa em sua integridade e dignidade e para contribuir no desenvolvimento da pesquisa dentro de

padrões éticos.
ANÁLISE: PENDÊNCIA ATENDIDA.

i) De acordo com a Resolução CNS nº 466/12, item IV.3.d, no TCLE, o pesquisador deve garantir que o participante da pesquisa possui o direito de retirar o consentimento sem sofrer qualquertipo de penalização. Solicita-se adequação.
RESPOSTA:...Foi-me garantido o sigilo das informações e que posso retirar meu consentimento a qualquer momento, sem que isto leve a qualquer penalidade ou interrupção de meu acompanhamento/assistência/tratamento.
ANÁLISE: PENDÊNCIA PARCIALMENTE ATENDIDA. Apesar de constar no texto a garantia do participante retirar seu consentimento a qualquer momento, o texto está escrito na primeira pessoa. Considerando que o TCLE é um documento onde todos os pontos e questões relativas à pesquisa que está sendo proposta serão apresentados pelo pesquisador aos participantes, estas informações devem ser registradas na terceira pessoa.
RECURSO:
Este item foi corrigido no TCLE:
O participante tem garantido o sigilo das informações e que pode retirar seu consentimento a qualquer momento, sem que isto leve a qualquer penalidade ou interrupção de seu acompanhamento/assistência/tratamento.
ANÁLISE: RECURSO ATENDIDO.

j) O Investigador Principal, ao assinar o TCLE, garante ao participante da pesquisa em seu nome e da Instituição de Pesquisa, todos os direitos elencados no documento. Assim sendo, ao "aluno" não cabe assinar o TCLE na qualidade de quem é responsável pela pesquisa. Portanto, solicita-se que o pesquisador responsável ou a pessoa por ele delegada deve assinar o TCLE. Solicita-se adequação
RESPOSTA: ...Eu, ____, abaixo assinado, fui informado e esclarecido pelo pesquisador ____ sobre a pesquisa, os procedimentos nela envolvidos, assim como os possíveis riscos e benefícios decorrentes de minha participação. Foi-me garantido o sigilo das informações e que posso retirar meu consentimento a qualquer momento, sem que isto leve a qualquer penalidade ou interrupção de meu acompanhamento/assistência/tratamento. Local e data ________________/_______/_______/__________/
Assinatura do sujeito (participante):

___________________________________ Assinatura do pesquisador responsável: ___________________________________

ANÁLISE: PENDÊNCIA ATENDIDA.

k) O TCLE deve informar o participante sobre as respostas as pendências 4 e 5, acima, diferenciando os riscos inerentes aos procedimentos operatórios do tratamento padrão (exodontia e colocação dos implantes) dos riscos inerentes aos procedimentos experimentais.

RESPOSTA: Os principais riscos operatórios inerentes e potenciais aos procedimentos cirúrgicos incluem, mas não são limitados a eles, os seguintes:

1.Desconforto pós-operatório e edema que pode necessitar de alguns dias de recuperação

2.Sangramento continuado que pode ser prolongado3. Infecção pós-operatória que pode exigir tratamento adicional.

4.Restrição da abertura bucal por alguns dias ou semanas.

5.Estiramento da abertura bucal por alguns dias ou semanas.

6.Fratura de mandíbula.

7.Injúria ao nervo subjacente aos dentes, resultando em adormecimento ou formigamento do lábio, queixo, bochechas, gengiva, dentes e/ou língua do lado operado, que pode persistir por semanas, meses ou, em raras circunstâncias, permanente.

8.Comunicação com o seio maxilar (uma cavidade normal localizada acima dos dentes superiores),exigindo cirurgia adicional.

9.Rejeição a materiais implantados (implantes, membranas, materiais sintéticos)

10.Deiscência de sutura expondo a área operada.

11.Não osseointegração dos implantes com a inutilização dos mesmos. Caso ocorra, estes implantes serãorepostos, ficando o CEPID responsável por esta reposição, porém não haverá de maneira alguma devolução

dos valores previamente combinados.

12.Necessidades de reintervenção cirúrgicas ou de um novo planejamento.

13.Os riscos inerentes aos procedimentos da coleta das amostras incluem apenas um aumento do desconforto pós-operatório, no momento da coleta 24 horas. Contudo o paciente já estará sob efeito de medicação analgésica.

ANÁLISE:PENDÊNCIA PARCIALMENTE ATENDIDA. Apesar dos riscos inerentes aos procedimentos operatórios (exodontia e colocação de implantes) terem sido elencados, estes foram relacionados

utilizando-se termos técnicos, o que dificulta o entendimento de uma pessoa leiga. Salienta-se ainda que os riscos inerentes aos procedimentos experimentais não constam do TCLE. Não está clara neste documento a questão da anonimização das amostras antes delas serem enviadas aos Estados Unidos.
RECURSO: Este item foi corrigido no TCLE:
“Antes de serem enviados à Universidade da Pennsylvania – EUA seus dados receberão um código com as inicias do primeiro e último nome a fim de preservar sua identidade”
“Os principais riscos da pesquisa incluem, mas não são limitados a eles, os seguintes:
1.Desconforto após a cirurgia e “inchaço” que pode necessitar de alguns dias de recuperação
2.Sangramento continuado que pode ser prolongado.
3.Contaminação após a cirurgia que pode exigir tratamento adicional.
4.Diminuição da abertura bucal por alguns dias ou semanas.
5.Pequeno corte no canto da boca por alguns dias ou semanas.
6.Fratura de mandíbula.
7.Trauma ao nervo próximo dos dentes, resultando em adormecimento ou formigamento do lábio, queixo, bochechas, gengiva, dentes e/ou língua do lado operado, que pode persistir por semanas, meses ou, em raras circunstâncias, permanente.
8.Comunicação com o seio maxilar (uma cavidade normal localizada acima dos dentes superiores), exigindo cirurgia adicional.
9.Rejeição a materiais (membranas, materiais sintéticos)
10.Afrouxamento da sutura “pontos” expondo a área operada.
11.Necessidades de outras cirurgias ou de um novo planejamento.
12.Os riscos dos procedimentos da coleta das amostras incluem apenas um aumento do desconforto após a cirurgia, no momento da coleta 24 horas. Contudo você já estará sob efeito de medicação analgésica.”
ANÁLISE: RECURSO ATENDIDO.

7) Quanto a BIOBANCO e BIORREPOSITÓRIO, não há esclarecimento quanto ao destino do material coletado para as análises de expressão gênica, se as mesmas serão armazenadas ou descartadas após as análises de expressão dos genes sugeridos no protocolo (página 9 de 13), BMP-4, BMP-7, TGF-beta, PDGF e GAPDH. Solicita-se esclarecimento.
RESPOSTA:...O material coletado para análise da expressão dos GENES BMP-4, BMP-7, TGF-BETA, PDGF E GAPDH será descartado no final do experimento, ou seja, no final da análise.

ANÁLISE: PENDÊNCIA ATENDIDA.

8) Os fitoquímicos a serem empregados são apresentados como produtos naturais. O Protocolo faz referência a alguns trabalhos de pesquisa onde os mesmos já estariam sendo pesquisados quanto a sua indicação como antiinflamatórios e/ou cicatrizantes. Ocorre que, diferente das tradicionais pesquisas com fármacos, não se apresentou uma brochura com relatos de pesquisas de avaliação de eficácia e segurança. Solicita-se adequação.
RESPOSTA: Seguem em anexo os artigos com as traduções.
ANÁLISE: PENDÊNCIA PARCIALMENTE ATENDIDA. Apenas um trabalho de cada produto proposto na metodologia foi inserido.
RECURSO:
Embora apenas um artigo de cada fitoquímico tenha sido anexado, observou-se que estes compostos naturais afetam osso não só pela sua capacidade não enzimática de criar ligações cruzadas no colágeno, mas também pela capacidade de alteração das respostas celulares como mudanças na disponibilidade de fatores de crescimento. E nenhum deles relatou qualquer interferência negativa ou prejudicial na saúde e nas respostas celulares. Resumindo, demonstraram-se como compostos seguros na utilização em humanos.
ANÁLISE: RECURSO ATENDIDO.

Situação do Parecer:
Aprovado

Considerações Finais a critério da CONEP:
Diante do exposto, a Comissão Nacional de Ética em Pesquisa - CONEP, de acordo com as atribuições definidas na Resolução CNS nº. 466 de 2012 e na Norma Operacional nº. 001 de 2013 do CNS, manifesta-se pela aprovação do projeto de pesquisa proposto.

Situação: Protocolo aprovado.

BRASILIA, 08 de Agosto de 2014

Assinado por:
Jorge Alves de Almeida Venancio
(Coordenador)

Background and Significance Today, it is estimated that one in four Americans suffer from bone loss in the form of pathologies of bone diseases such as osteogenesis imperfecta, Paget's, osteoporosis and periodontal disease. In the craniofacial arena, periodontal disease is most often the cause of bone loss. The periodontium is one of the most challenging areas for bone regeneration due to the complexity of the cell population. Finding ways to prevent this disease and thus loss of bone mass as well as to regenerate bone predictably are critical. While there are multiple factors that contribute to formation and maintenance of bone, a common point of regulation is the availability of appropriate growth factors. In addition to growth factors, the physical environment of cells is important for tissue maintenance. Matrix stiffness, for instance, can direct cell lineage decisions and make differentiation efficient (1). Furthermore, mineralization does not properly occur when the collagen scaffold is poorly formed, which result in weak bones (2). A well-organized matrix with proper cross-linkage can be more resistant to degradation and provide appropriate support to cells. Thus, the extracellular matrix (ECM) is a significant factor for promoting bone health. An emerging strategy for enhancing bone health is to utilize naturally derived compounds that can potentially change matrix quality and cell responses. Several compounds, which are present in plants and foods, have been indicated for a wide range of health problems from inflammation to bacteremia to cancer (3-6). Many natural compounds are known to have positive effects on collagen strength (7-9), tissue mass (10, 11) and cell proliferation (12, 13). Of those compounds, three have shown to reduce collagen digestibility – grape seed extract (containing proanthocyanidin, PA), gardenia plant extract (containing genipin, GE) and grapefruit extract (containing hesperidin, HE and naringenin, NA) (79, 14). However, their effects on matrix cross-linking and organization as well as cell function have not been clearly investigated. Based on the above reported positive effects, the first molecular mechanisms we would like to investigate are their effects on growth factor expression and bone matrix quality. Those can significantly affect long-term bone formation. While there is abundant support for recommending diets rich in nutrients from fruits and vegetables, there is only limited research evidence that phytochemicals may have any physiological effect. This is especially because of their low bioavailability after intake (15). The understanding of how increased bioavailability (through supplementation) would affect the human body and the mechanisms of action are limited and further research on their biological activities is warranted. Among the most consumed phytochemical supplements are the phenolic compounds (from fruits and vegetables) and the terpins (from plants). Phenolic compounds or flavonoids are sub-classified as anthocyanidins, flavonols, flavanones, flavanols, flavones and isoflavones (16). One of the most popular commercially available flavonoids is grape seed extract, which is a mixture of flavanols named proanthocyanidin (PA) monomers and polymers (also in apples, berries, red wine and chocolate). Grape seed extract has been widely used with the promise to prevent inflammation, protect the cardiovascular system, and be antimicrobial (16-18). Previously, we have shown that PA is an effective type I collagen cross-linker, but it does not effectively prevent bacterial colonization of dentin (8). Another popular commercially available phenolic compound is flavanone (present in citrus fruits). HE and NA, both flavanones in the grapefruit extract, have been studied in cells. HE has been shown to stimulate osteoblasts differentiation through activation of bone morphogenetic protein (BMP) signaling (19) while NA has been shown to improve bone mass in rats via intra-gastric injections (11). The other popular group of phytochemicals for health applications is the terpins. In that group, genipin (GE), which is derived from the fruit Gardenia Jasminoides Ellis, may be the compound most extensively studied. Research in this compound has mainly focused in its ability to cross-link bioscaffolds, despite its wide use in hypertension and diabetes prevention (possible anti-inflammatory function) (13, 14, 20). The use of PA, GE and HE/NA

for bone strength and slower breakdown (through cross-linking collagen) and for improved cell function (growth factor availability) could potentially be applied to many physiological and pathological conditions leading to better function and quality of life to many. In the field of periodontology, phytochemicals could lead to improved bone remodeling, halting of periodontal disease through matrix stiffening, secretion of growth factors, and many other functions still not unveiled.

Methods This translational study is designed as follows: In aim 1, we will examine the effects of ingesting PA, GE, and HE/NA on gene expression of growth factors that are critical for bone maintenance and repair following tooth extraction by using quantitative real time-PCR. In aim 2, we will determine whether continued systemic exposure to the compounds during healing of the extraction site lead to high quality bone formation by using histology to observe overall morphometry and collagen organization. Approval from the appropriate institutional review board of the CONEP (Comissao Nacional de Etica em Pesquisa) will be obtained for human studies. Each treatment [PA, GE, NA/HE, and no treatment (NTx)] will be assigned to implant sites. Patients recruited at the Implant center CEPID in need of implant placement will be enrolled and randomly assigned to treatment groups. To participate in the study, participants should be 18 years of age or older and males. Participants should be non-smokers, have no periodontal disease, and have no medical condition that contraindicates implant therapy (exclusion criteria). Subjects will start ingesting at least 125 mg t.i.d. of the phytochemicals (based on manufacturer's recommendation) (Grape Seed Extract [PA], Gardenia Extract [GE], and Grapefruit Extract [NA/HE], Vitamin Shoppe, NJ, USA) two weeks prior to extraction and maintain this regimen for eight weeks after this first surgical procedure (Total of 10 weeks). The extraction will take place with careful technique to preserve the socket. After extensive curetting and irrigation of the alveolar socket, hemostasis will be obtained and the site covered and sutured to isolate the wound from the oral cavity. Aim 1: At 24h of tooth extraction, under anesthesia, the underlying tissue and a core of the soft tissue within the socket will be extracted with a 4mm-tissue punch (Salvin, NC, USA). The site will be recovered with Collaplug (Zimmer). The biopsied specimen will be immediately placed in RNAlater (Qiagen, CA, USA) for ease of transportation. The RNA will be extracted with TRIzol reagent (Invitrogen, California, USA) and 2 µg of total RNA will be converted into cDNA by using the Omniscript reverse transcriptase kit (Qiagen). Quantitative real time PCR (qRTPCR) will be performed using the sequence-specific primers for the growth factors BMP-4 (Hs01041266_m1, Applied Biosystems, CA, USA), BMP-7 (Hs00233476_m1, Applied Biosystems), TGF-β (Hs00998133_m1, Applied Biosystems) and PDGF (Hs00964426_m1, Applied Biosystems) or wound healing array. The reactions will be prepared and analyzed in triplicates by Applied Biosystems StepOnePlusTM (Applied Biosystems). The mRNA expression level of the genes of interest will be calculated relative to glyceraldehyde-3-dehydrogenase (GAPDH) ([illegible]CT method (21). Subjects will be followed up for healing assessment. Aim 2: At day 60 of tooth extraction, under anesthesia, a full thickness flap will be raised and a 3-mm trephine core obtained at the site prior to implant placement. The specimen will be placed in 10% buffered formalin for three days and then demineralized with 0.5 M EDTA pH 7.4 for four weeks. The samples will be dehydrated in several ascending gradations of ethanol (50-100%), soaked in xylene and embedded in paraffin. Six micrometer sections from each paraffin block will be cut using a microtome Leica Jung RM2045 (Leica, Germany). Sections will be stained for histomorphometry, osteoclast activity and by picrosirius red staining (PSR) to evaluate collagen organization and maturation . Quantification of colors of PSR will be done by Image J software (NIH, MA, USA). For PSR, under polarized light in a light

microscopic setting, yellow to green color represent lose, immature, and disorganized collagen matrix while orange to red represent well-packed, mature, and organized collagen (22).

References

1. Engler AJ, Sen S, Sweeney HL, Discher DE. Matrix elasticity directs stem cell lineage specification. Cell. 2006;126(4):677-89. Epub 2006/08/23.

2. Buxton PG, Bitar M, Gellynck K, Parkar M, Brown RA, Young AM, et al. Dense collagen matrix accelerates osteogenic differentiation and rescues the apoptotic response to MMP inhibition. Bone. 2008;43(2):377-85. Epub 2008/05/27.

3. Jain M, Parmar HS. Evaluation of antioxidative and anti-inflammatory potential of hesperidin and naringin on the rat air pouch model of inflammation. Inflammation research : official journal of the European Histamine Research Society [et al]. 2011;60(5):483-91. Epub 2010/12/25.

4. Nam KN, Choi YS, Jung HJ, Park GH, Park JM, Moon SK, et al. Genipin inhibits the inflammatory response of rat brain microglial cells. International immunopharmacology. 2010;10(4):493-9. Epub 2010/02/04.

5. Engelbrecht AM, Mattheyse M, Ellis B, Loos B, Thomas M, Smith R, et al. Proanthocyanidin from grape seeds inactivates the PI3-kinase/PKB pathway and induces apoptosis in a colon cancer cell line. Cancer letters. 2007;258(1):144-53. Epub 2007/10/10.

6. Neto CC. Cranberry and its phytochemicals: a review of in vitro anticancer studies. The Journal of nutrition. 2007;137(1 Suppl):186S-93S. Epub 2006/12/22.

7. Han B, Jaurequi J, Tang BW, Nimni ME. Proanthocyanidin: a natural crosslinking reagent for stabilizing collagen matrices. Journal of biomedical materials research Part A. 2003;65(1):118-24. Epub 2003/03/14.

8. Walter R, Miguez PA, Arnold RR, Pereira PN, Duarte WR, Yamauchi M. Effects of natural cross-linkers on the stability of dentin collagen and the inhibition of root caries in vitro. Caries research. 2008;42(4):263-8. Epub 2008/06/05.

9. Sung HW, Chang Y, Chiu CT, Chen CN, Liang HC. Crosslinking characteristics and mechanical properties of a bovine pericardium fixed with a naturally occurring crosslinking agent. Journal of biomedical materials research. 1999;47(2):116-26. Epub 1999/08/17.

10. Stipcevic T, Piljac J, Vanden Berghe D. Effect of different flavonoids on collagen synthesis in human fibroblasts. Plant foods for human nutrition (Dordrecht, Netherlands). 2006;61(1):29-34. Epub 2006/04/28.

11. Swarnkar G, Sharan K, Siddiqui JA, Mishra JS, Khan K, Khan MP, et al. Identification of a Rare Naringenin Analog from a Medicinal Plant having Potent Bone Anabolic Effect by Acting as an Osteoblast Oestrogen Mimic. British journal of pharmacology. 2011. Epub 2011/08/26.

12. Takahashi T, Kamiya T, Yokoo Y. Proanthocyanidins from grape seeds promote proliferation of mouse hair follicle cells in vitro and convert hair cycle in vivo. Acta dermato-venereologica. 1998;78(6):428-32. Epub 1998/12/02.

13. Sisson K, Zhang C, Farach-Carson MC, Chase DB, Rabolt JF. Evaluation of cross-linking methods for electrospun gelatin on cell growth and viability. Biomacromolecules. 2009;10(7):1675-80. Epub 2009/05/22.

14. Liang HC, Chang Y, Hsu CK, Lee MH, Sung HW. Effects of crosslinking degree of an acellular biological tissue on its tissue regeneration pattern. Biomaterials. 2004;25(17):3541-52. Epub 2004/03/17.

15. Scalbert A, Williamson G. Dietary intake and bioavailability of polyphenols. The Journal of nutrition. 2000;130(8S Suppl):2073S-85S. Epub 2000/08/05.

16. Teixeira S. Bioflavonoids: proanthocyanidins and quercetin and their potential roles in treating musculoskeletal conditions. The Journal of orthopaedic and sports physical therapy. 2002;32(7):357-63. Epub 2002/07/13.

17. Howell AB. Cranberry proanthocyanidins and the maintenance of urinary tract health. Critical reviews in food science and nutrition. 2002;42(3 Suppl):273-8. Epub 2002/06/13.

18. Bagchi D, Sen CK, Ray SD, Das DK, Bagchi M, Preuss HG, et al. Molecular mechanisms of cardioprotection by a novel grape seed proanthocyanidin extract. Mutation research. 2003;523-524:87-97. Epub 2003/03/12.

19. Trzeciakiewicz A, Habauzit V, Horcajada MN. When nutrition interacts with osteoblast function: molecular mechanisms of polyphenols. Nutrition research reviews. 2009;22(1):68-81. Epub 2009/02/27.

20. Sundararaghavan HG, Monteiro GA, Lapin NA, Chabal YJ, Miksan JR, Shreiber DI. Genipin-induced changes in collagen gels: correlation of mechanical properties to fluorescence. Journal of biomedical materials research Part A. 2008;87(2):308-20. Epub 2008/01/09.

21. Livak KJ, Schmittgen TD. Analysis of relative gene expression data using real-time quantitative PCR and the 2(Delta Delta C(T)) Method. Methods (San Diego, Calif). 2001;25(4):402-8. Epub 2002/02/16.

22. Junqueira LC, Bignolas G, Brentani RR. Picrosirius staining plus polarization microscopy, a specific method for collagen detection in tissue sections. Histochem J. 1979;11(4):447-55.
